# The Prevalence of OCD Like Symptoms Among UAE Residents During COVID-19 Pandemic

**DOI:** 10.1192/bjo.2022.78

**Published:** 2022-06-20

**Authors:** Hamid Alhaj, Amena Ali, Hamid Askari, Hend Ibrahim, Leena Mohamed Ali, Leen Kassasand, Noura Al Nasere

**Affiliations:** 1 University of Sharjah, Sharjah, UAE; 2 University Hospital Sharjah, Sharjah, UAE

## Abstract

**Aims:**

Obsessive-compulsive disorder (OCD) is a psychiatric disorder characterised by repetitive, undesirable thoughts or acts. With the pandemic being on the rise, public health authorities have urged people to take proper health measures, specifically around hand washing and social distancing. It is hypothesised that such sudden change would cause excessive hygiene habits in people predisposed to OCD. Our study aimed to measure how COVID-19 pandemic affected the prevalence of OCD-like symptoms among the UAE population. Furthermore, we explored the correlation between these symptoms and demographic factors.

**Methods:**

This was a quantitative cross-sectional study that used an online survey platform to collect responses from male and female adult UAE residents (aged between 18 and 60 years). The self-administered questionnaire included Yale-Brown Obsessive compulsive scale (YBCOS) to test the severity of obsessions and compulsions, in addition to demographic questions. People with a previous diagnosis of obsessive-compulsive disorder were excluded. Data were analysed using SPSS 23. A bivariate analysis to examine the correlation between the severity of the reported symptoms and sociodemographic characteristics, age, and ethnicity was conducted. A p-value <0.05 was considered statistically significant.

**Results:**

A total of 343 questionnaires were completed and used for analysis; 244 of which were females and 99 were males. 63.5% of females had no symptoms of OCD, 24.6% had mild symptoms, 9% had moderate symptoms, 2.5% had severe symptoms, and 0.4% had extreme OCD symptoms. In males, 75.8% had no OCD symptoms, 18.2% had mild OCD, 6.1% had moderate OCD, and 0% had severe or extreme OCD. A significant correlation was found between the Emirate of residence and the severity of OCD-like-symptoms (P = 0.042). The most significant scores of OCD symptoms were in Sharjah, with 10 people out of 108 displaying moderate to extreme symptoms, Abu Dhabi with 9 out of 115 and Fujairah with 7 out of 56. Level of education, occupation and age had no significant role in the exhibition of the symptoms.

**Conclusion:**

To our knowledge this is the first study to investigate the prevalence of OCD-like symptoms within the UAE residents during COVID-19 pandemic. The symptoms of potential clinical significance of OCD are high, especially in females compared to the global prevalence studies prior to COVID-19, although a within-subject comparison is not possible. Further research is warranted to investigate the long-term effect of COVID-19 on OCD-like and other neuropsychiatric symptoms and elucidate possible mechanisms.

